# Supplementary material for: Investigating traditional and novel predictors of a single versus multiple fragility fractures in a large observational cohort
Source: PLoS One. 2026 Feb 23;21(2):e0343413. doi: 10.1371/journal.pone.0343413 (PMC12928489; doi:10.1371/journal.pone.0343413)
Supplement: Attachment — Submitted filename: rebuttal letter .docx [file pone.0343413.s001.docx]

Dear Editors,

I would first like to thank you both for taking time out of your busy schedule to review our manuscript. From reading your comments it is clear that significant time was taken to review our papers and you have provided us with constructive comments which we have endeavoured to address.

Some general changes made to our paper:

- We have updated the definition of osteoporosis to include bone microarchitecture
- We have removed hypothyroidism as a secondary cause of osteoporosis from our models, which has affected our results ever so slightly. Though, this has not affected the conclusions of our paper
- We have added a additional paragraph in our discussion to talk about the implications of our paper in the context of current fracture calculators
- Clarification of reference levels used in our models

Below you will find a point-by-point response to your comments:

| **Reviewer's comment:** | **Our response:** |
| --- | --- |
| **Reviewer 1:** |  |
| OVERALL ASSESSMENT: This is a methodologically sound observational study utilizing a large real-world DXA cohort (n=43,801) to identify predictors of multiple versus single fragility fractures. The GAM analysis is appropriate, confirming traditional risk factors while uncovering a novel alcohol-falls risk interaction (OR 7.60, 95% CI 2.77-20.9) with clear clinical implications. Particularly interesting is the potential role of PBF% as a novel body composition predictor. The study addresses an important gap, as literature specifically examining multiple fracture predictors remains limited. | We thank the reviewer for taking the time to review our manuscript and providing constructive comments. Additionally, we are happy that the reviewer found our study interesting and methodologically sound. |
| REQUIRED MINOR REVISIONS:  1. ABSTRACT - BACKGROUND: Please add 1-2 sentences providing brief osteoporosis context, defining it as a chronic systemic disease characterized by reduced bone density, increased fragility fracture risk, and severe clinical consequences (morbidity, mortality, substantial healthcare costs). | We have updated our definition of osteoporosis to also define it as a loss of microarchitecture. This has been updated in both the abstract and the introduction.  We have also discussed the relationship between osteoporosis and fragility fractures in both the abstract and introduction. |
| 2. METHODS - Data Collection: Remove hypothyroidism from the secondary osteoporosis causes list, as the literature does not consistently support it as a fracture risk factor. Indeed, I suggest to remove the patients with hypotyroidism from the secondary osteoporosis group. | As requested, we have removed hypothyroidism and agree with you that this is probably an inconsistent cause of fragility fractures. This is further evidenced by the fact that FRAX also does not list this as a secondly in their guidance when using frax.  The removal of hypothyroidism only made a small difference to our regression parameters and p-values which have been updated accordingly. The conclusions of our paper remain the same. |
| 3. DISCUSSION - Body Composition: Add a limitation statement clarifying that PBF% represents a partial measure (derived from lumbar spine + bilateral femurs only) rather than validated total body composition analysis. This warrants cautious interpretation of findings and prospective validation in future studies. | We agree that this is not a clinically validated measure of body composition but rather a novel one. We have made this clear in our updated discussion.  Moreover, our group is currently working on acquiring data that looks at the correlation between partial and total body fat measures in the hopes to trying to validate our measure described in the paper. Though this is a separate study. |
| MINOR TECHNICAL POINTS - Table 2: Please clarify the reference category for the alcohol × falls risk interaction terms | This was an oversight and have now clarified the reference group both in the results section and the table heading.  The reference group is ‘no’ and ‘no’ for interaction effects. For other binary variables in our model the reference category is also ‘no’, which we have also updated accordingly. |
| - Discussion: Consider briefly acknowledging that the cross-sectional design limits causal inferences | We appreciate this is an important point and all results should be interpreted in this context. Our strengths and limitations section is very transparent and we have reported the cross sectional design of our study being a limitation. More specifically we say ‘Additionally, the cross-sectional nature of our study (data collected only at the time of the first scan) limits our ability to draw causal conclusions. Given that our study involved a high-risk referred population, it may not reflect the general population and could be subject to sampling bias.’  To avoid our paper being repetitive and disrupting the overall flow of our paper we will not repeat this point in the discussion section as it is discussed in the strengths and limitations section as described. |
| **Reviewer 2:** |  |
| The authors have conducted a study addressing an important and clinically relevant question, namely the identification of predictors distinguishing single from multiple fragility fractures in a large observational cohort. The manuscript is generally clear, methodologically sound, and the conclusions are largely supported by the presented data. I believe the study is suitable for publication after minor revisions. | We appreciate the reviewer taking the time out of their busy schedules to review our manuscript and provide us with constructive comments. |
| - Introduction. The authors state that osteoporosis is defined by the World Health Organization (WHO) as a reduction in bone density of 2.5 standard deviations below that of a young healthy adult population, measured at the femoral neck. This definition could be reformulated to better reflect the original WHO reference by incorporating both microarchitectural alterations and a more precise densitometric description. | We agree that our definition of osteoporosis needs updating as also highlighted by reviewer 1. We have now revised our definition to also include microarchitectural distortion of bone as measured by the trabecular bone score. |
| - Results. It may be appropriate to include a study flow chart as the first figure in the Results section to clearly illustrate the number of subjects initially screened and those ultimately included in the final analysis. | To clarify all patients who were scanned at our scanner and reported multiple fragility fractures were eligible for inclusion in our study as described in our statistical analysis section of our paper.  Given this we believe that a flow chart is unlikely to be useful or informative, hence, why we have opted not to include this. Additionally, our methods also clearly report the total number of referrals as well as thought reporting 1 or more fragility fractures.  We hope this clarifies. |
| - Results. Many of the predictors of multiple fragility fractures identified in this study are already included in FRAX or FRAXplus, which represent established tools for the simultaneous integration of multiple clinical risk factors. It would therefore be of interest to assess whether differences in estimated fracture probability exist between individuals reporting a single versus multiple fragility fractures, and whether a specific probability threshold could be identified to discriminate between these two groups. | We do agree that many of our predictors are indeed in FRAX. Our paper novelty is that our study has found a interaction effect between alcohol and falls risk which we believe is not being accounted for accurately in fracture calculators.  With that being said the reason why it is not feasible to compare the frax score, though a completely valid suggestion is because the FRAX score was not directly inputted into our database by the technician at the appointment. We could have endeavoured to calculate the frax score using a web scraping algorithm / using a published algorithm like q fracture however, unfortunately FRAX is proprietary and quite difficult to work with. Hence why this is not possible  However, we have now added a paragraph in our discussion talking about the implications of our results in the context of FRAX and have made suggestions for future research. |
| - Results. The manuscript does not include information on osteoporosis-specific treatments (e.g. antiresorptive or anabolic therapies), which may substantially influence fracture risk and the occurrence of multiple fragility fractures. The authors should clarify whether treatment data were unavailable or not collected, and acknowledge this as a limitation, as prior or ongoing therapy could confound the observed associations. | This is a valid point and we do believe this would be a important line of enquiry in regards to exploring factors associated with multiple fragility fracture. It would be interesting to know how medications modify this relationship. Given we do not have this data we have mentioned this as a limitation of our study in the strengths and limitation section of our paper. |
| - Results. The very high odds ratio observed for the interaction between excessive alcohol consumption and falls risk may be influenced by the relatively small number of individuals in this subgroup. This is also reflected by the wide confidence intervals. The authors are encouraged to acknowledge this limitation explicitly in the Results or Discussion | As you have quite rightly pointed our this is likely a sample size issue which we have discussed transparently in our discussion section. |
| - General comment. For consistency and clarity, all p-values should be reported to three decimal places throughout the text and tables. When p-values are smaller than this threshold, they should be reported uniformly as p<0.001. | We have now reported all p values to 3 dp as requested or p<0.001. |

Given the changes we have made we feel that our study will now be ready for publication. We hope the reviewers feel the same.

Many thanks,

Hamzah Amin

(on behalf of authors)
